# The mediating effect of traditional Chinese medicine (TCM) health literacy between TCM culture promotion and residents' health status

**DOI:** 10.3389/fpubh.2024.1386085

**Published:** 2024-08-01

**Authors:** Xiaofan Zhang, Xiang Hong, Zongming Zhang

**Affiliations:** ^1^Institute of Literature in Chinese Medicine, Nanjing University of Traditional Chinese Medicine, Nanjing, China; ^2^Department of Epidemiology and Health Statistics, School of Public Health, Southeast University, Nanjing, China

**Keywords:** health status, mediating effect, traditional Chinese medicine culture propaganda, traditional Chinese medicine health literacy, Chinese medicine

## Abstract

**Background:**

Traditional Chinese medicine (TCM) is a medical system with a long history and unique theories and techniques, playing a crucial role in maintaining and promoting human health. Disseminating TCM cultural knowledge is essential for enhancing the TCM health literacy and health status of people. This study aimed to investigate how TCM health literacy mediated the relationship between the TCM cultural ambiance and people's health status.

**Methods:**

A multi-stage random sampling method was employed to select 22,125 permanent residents in Jiangsu Province for a survey aimed at examining the popularization of TCM culture. The survey categorized the intensity of TCM cultural ambiance into four grades (0–3) based on the promotion activities in various settings, including street/community, medical service institutions, and educational/work institutions. Furthermore, the survey evaluated TCM health literacy, overall health status, and prevalence of chronic diseases using the 2017 Chinese Citizens TCM Health Literacy Survey Questionnaire. The regression analysis was used to examine the correlation between the TCM cultural ambiance and TCM health literacy and health status. Also, a mediation model was applied to explore the mediating effect of TCM health literacy on the TCM cultural ambiance and the public's self-assessment of health and reporting of chronic diseases.

**Results:**

The rate of popularization of TCM culture was 79.3% in Jiangsu Province, with a TCM health literacy level of 14.76%. The average score for public self-assessment of health was 37.80 ± 9.70, and the prevalence of chronic diseases was reported at 32.66%. A positive correlation was observed between cultural promotion ambiance and TCM health literacy. The impact of the cultural promotion ambiance on health literacy also increased with the increase in promotion grade (*P* < 0.001). The correlation analysis showed no statistically significant correlation of promotion grade 1 [β = 0.21, 95% confidence interval (CI): −0.26 to 0.67] with the health status of residents, whereas promotion grades 2 (β = 1.47, 95% CI: 1.12–1.83) and 3 (β = 4.61, 95% CI: 4.19–5.04) were positively correlated with self-health evaluation. TCM health literacy was positively correlated with self-health evaluation (β = 2.10, 95% CI: 1.72–2.48) and negatively correlated with the risk of chronic diseases (odds ratio = 0.53, 95% CI: 0.48–0.58). The analysis of the mediating effect revealed that the TCM cultural ambiance directly impacted self-health evaluation and the prevalence of chronic diseases, with coefficients of 1.131 and −0.027 (*P* < 0.001), respectively. TCM health literacy had a significant mediating effect on the relationship between the cultural promotion ambiance and self-health evaluation, as well as with the prevalence of chronic diseases (*P* < 0.001). The mediating effect accounted for 17.8% and 42.2% of the total effect, respectively.

**Conclusions:**

TCM health literacy served as a mediating factor in the positive relationship between the TCM cultural ambiance and health status. Theoretically, it can improve the overall health of residents by intensifying the dissemination of TCM culture and enhancing their health literacy.

## 1 Introduction

Traditional Chinese medicine (TCM) encompasses the medical practices of various ethnic groups in China, including the Han nationality and ethnic minorities. This medical system embodies the perspectives of Chinese people on life, health, and illness, and is characterized by its extensive historical legacy, traditional customs, and distinctive theories and methodologies (1). Research has demonstrated that TCM utilization was linked to the management of infectious diseases, early intervention, successful treatment, slowing down disease progression, and facilitation of recovery from chronic and severe illnesses (2–6). As an essential part of China's cultural heritage, TCM has been recognized as an intangible cultural heritage by the United Nations Educational, Scientific and Cultural Organization (7). The Chinese government has elevated the development of TCM to the national strategic level, emphasizing that improving residents' health is contingent upon enhancing their health literacy, particularly in TCM (8).

Although research on TCM has been continuously expanding and evolving, no universally accepted definition for TCM health literacy has been established to date. In 2009, the notion of “public TCM literacy” was introduced, highlighting the significance of comprehending TCM theory and philosophy (9). In 2014, a group of researchers proposed a definition for “TCM health care literacy,” which centered on the independent selection and application of TCM by individuals (10). Some scholars contend that TCM not only encompasses natural science but also embodies the elements of humanities. Hence, they have proposed the concept of “TCM health cultural literacy” as an extension of “TCM health care literacy.” It also encompasses the comprehension level of TCM culture (11). In 2016, the research team from the China Administration of Traditional Chinese Medicine and Beijing University of Chinese Medicine developed the Questionnaire of Chinese Citizens' Health Literacy of Traditional Chinese Medicine in accordance with this concept. The questionnaire demonstrated strong validity and reliability, and has been widely disseminated across the country to evaluate the TCM health literacy of the populace (12).

Enhancing health literacy is essential for advancing public health, as evidenced by the correlation between health literacy and health-related behaviors (13, 14). Effective communication of health information is pivotal in enhancing public health literacy. Friis et al. demonstrated that health literacy, specifically the ability to comprehend health information, had a mediating effect on the correlation between educational attainment and health behaviors (15). Zhu Bing et al. found that the implementation of a TCM cultural environment, including the utilization of bulletin boards, TCM health tour activities, and dissemination of information through newspapers, television, WeChat, and other media, resulted in an overall enhancement of TCM health literacy in Anhui Province, China (16). Presently, the general public has access to health-related information through various channels, such as community centers, educational institutions, workplaces, medical service institutions, and other platforms. The dissemination of health knowledge primarily occurs through bulletin boards, printed materials, audio-visual resources, and lectures (17–19). Various levels of TCM culture promotion may lead to a varying degree of TCM culture ambiance, resulting in differences in health literacy. However, the evidence indicating that the TCM health literacy of residents mediates the relationship between the ambiance promoting TCM culture and the health status of residents is lacking. Therefore, this study was based on the National Administration of Traditional Chinese Medicine Science and Technology Fund Project to assess the current situation of the popularization of TCM culture and the level of health literacy among residents in Jiangsu Province from 2017 to 2022, and to analyze the relationship between the ambiance promoting TCM culture and TCM health literacy. The mediating effect model was used to explore the mediating effect of TCM health literacy on the cultural promotion ambiance, public self-health evaluation, and chronic disease reporting.

## 2 Materials and methods

### 2.1 Participants

The inclusion criteria for participants were as follows: residents aged 15–69 years in Jiangsu Province from 2017 to 2022, living in Jiangsu Province for more than 6 months in the last 12 months, and able to independently complete the questionnaire or with the assistance of researchers. The exclusion criteria were as follows: residents living in military bases, hospitals, prisons, nursing homes, dormitories, and so forth; those unwilling to be investigated; and those with cognitive impairment. All participants have signed informed consent forms. Minor participants can participate only with the consent of their guardians. The sample size estimation formula for cross-sectional studies is used. Based on the pre-survey results, the qualification rate of TCM literacy is approximately 15%. Our allowable error is set at 1%, with a Type I error set at 0.05. Considering that this study is using multi-stage random sampling, we have set the design effect (Deff) at 16. Therefore, this study needs to include at least 18,384 participants.

### 2.2 Design

This study used a multi-stage random sampling method combined with probability proportionate to size two-stage cluster sampling to randomly select 14 monitoring counties (cities and districts) in Jiangsu Province, and three streets (towns) were randomly selected from each monitoring point. Furthermore, two communities (villages) were chosen from the selected streets (towns), and 55 families were randomly selected from the selected communities (villages) by the simple random sampling method. Finally, one permanent resident aged 15–69 years from each household was selected as the survey object. In summary, a total of 22,125 questionnaires were distributed in this study, after excluding some questionnaires with poor quality and people who withdrew from informed consent, there were 19,648 valid questionnaires remaining.

### 2.3 Definition of TCM health literacy

The 2017 Chinese Citizens Health Literacy of Traditional Chinese Medicine Questionnaire, developed by the State Administration of Traditional Chinese Medicine, was used in this study. It had the same content as the 2016 version. The questionnaire demonstrated strong reliability and effectively represented health literacy related to Chinese medicine, as indicated by a Cronbach's coefficient of 0.809 and an expert validity value of 0.786 (20).

The survey included 37 questions worth 100 points, which were divided into 5 categories: judgment, single-choice, multiple-choice, material-analysis, and basic information. Correct answers to judgment and single-choice questions earned one point, whereas incorrect answers received zero point. For multiple-choice questions, two points were given for selecting completely correct answer options and zero point for choosing the wrong answer or not answering. The scoring system for material-analysis questions was the same as for single- and multiple-choice questions. If participants scored 70 points or higher, they were considered to have a basic understanding of TCM health literacy.

### 2.4 Definition of TCM cultural ambiance

The TCM cultural ambiance could be classified into three types based on the different settings in which it was promoted: public (township/village) propaganda, medical service institution propaganda, and school/workplace propaganda. The intensity of the ambiance promoting TCM culture was divided into four levels (0–3) based on the extent of promotional activities in these areas.

### 2.5 Self-assessment of health level

The participants' chronic disease status and self-perceived level of health were assessed through self-reported responses. Chronic diseases mentioned in the questionnaire included hypertension, heart disease, cerebrovascular disease, diabetes, and malignant tumors. Any self-reported presence of these conditions was classified as indicative of a chronic disease. Additionally, the respondents were asked to self-assess their physical health status over the past year with a score of 50 representing optimal health and 0 representing the poorest health.

### 2.6 Statistical analysis

The statistical analysis was conducted using R software version 4.1.3. Continuous variables were presented as mean ± standard deviation, and categorical variables were presented as frequency/proportion. Multiple linear regression was employed to examine the relationship between the TCM cultural ambiance and health literacy scores and self-health evaluation, as well as the correlation between categorized health literacy and self-health evaluation. Additionally, unconditional logistic regression was used to investigate the influence of promotional ambiance on categorized health literacy and reporting of chronic diseases, as well as the impact of categorized health literacy on reporting of chronic diseases. In these multiple regression models, we adjusted age, sex, education level, occupation, and annual income for potential confounding factors control. The R software's mediation package was used to analyze the mediating effect of TCM health literacy on the relationship between the TCM cultural ambiance and self-health assessment. The significance level for the test was set at α = 0.05.

## 3 Results

### 3.1 General situation

A survey was conducted in Jiangsu Province involving 22,125 residents. Furthermore, 19,648 valid questionnaires were obtained, resulting in an 88.8% response rate. The average age of the participants was 50 years, with 44.4% being male and 55.6% female. Among the participants, 20.9% had a secondary school education or lower, 38.6% had a middle school education, 21.6% had a senior high school education, and 18.9% had education beyond senior high school. Migrant workers represented 30.0% of the participants, whereas regular workers and staff accounted for 15.3% and 18.9%, respectively. The majority of households had an annual income ranging from 20,000 to 80,000 Yuan (44.6%), followed by 80,000–200,000 Yuan (24.5%) and < 20,000 Yuan (24.0%). Basic demographic details are provided in Table 1.

**Table 1:** Basic demographic information.

	***N***	**%**
Age, mean ± SD	49.61 ± 12.81	
**Gender**		
Male	8,715	44.4
Female	10,933	55.6
**Educational level**		
Primary school and below	4,110	20.9
Middle school	7,575	38.6
High school	4,251	21.6
Higher education and above	3,712	18.9
**Occupation**		
Farmer	5,893	30.0
Worker	3,012	15.3
Clerk	3,717	18.9
Others	7,026	35.8
**Annual income/year, RMB**		
< 20,000 (< $2,973^#^)	4,715	24.0
~80,000 (~$11,894)	8,760	44.6
~200,000(~$29,730)	4,809	24.5
≥200,000 (≥$29,730)	1,364	6.9
**Promotional venues for TCM culture**		
Street/community	13,619	69.31%
Medical service institutions	14,552	74.06%
Work units/Schools	4,021	20.46%
**TCM health literacy**, mean ± SD	48.95 ± 18.38	
Adequate TCM health literacy	2,900	14.76%
Chronic diseases	6,418	32.66%
Self-assessed health score, mean ± SD	37.80 ± 9.70	

^#^The questionnaire is in Chinese, and the exchange rate between Chinese Yuan and US Dollar is converted based on the average exchange rate for the year 2022. 1 US Dollar = 6.7261 Chinese Yuan.

Among the participants, 13,619 (69.31%) reported that TCM culture was promoted in the street/community (township/village), 14,552 (74.06%) reported that TCM culture was promoted by medical service institutions, and 4,021 (20.46%) reported that TCM culture was promoted by work units/schools; the total propaganda rate was 79.3%. According to the intensity of propaganda, 4,058 people (20.65%) reported a level of 0, 2,672 people (13.60%) reported a level of 1, 9,234 people (47.00%) reported a level of 2, and 3,684 people (18.75%) reported a level of 3.

The average TCM health literacy score for residents aged 15–69 years in Jiangsu Province was 48.95 ± 18.38. Adequate health literacy was defined as a score of 70 points or higher, and 2,900 residents (14.76%) [95% confidence interval (CI): 14.27%−15.26%] met this standard. A total of 6,418 chronic diseases were reported, indicating a prevalence rate of 32.66%. The average self-assessed health score was 37.80 ± 9.70.

### 3.2 Relationship between the TCM cultural ambiance and TCM health literacy

The study reported a significant correlation between the TCM cultural ambiance and TCM health literacy (*P* < 0.001). Compared with the lowest promotion grade, the average health literacy score increased by 7.92 points for grade 1 (β = 7.92, 95% CI: 7.08–8.76), by 11.09 points for grade 2 (β = 11.09, 95% CI: 10.45–11.72), and by 20.09 points for grade 3 (β = 20.09, 95% CI: 19.32–20.86). Even after adjusting for age, sex, education level, occupation, and annual income, the correlation remained statistically significant (*P* < 0.001). Additionally, the impact of the promotional ambiance on health literacy became more pronounced with the increase in the level of promotion. Taking 70 grades as the cut-off value of health literacy, compared with promotion grade 0, the probability of having health literacy in grade 1 was 2.11 times that of the reference level (OR = 2.11, 95% CI: 1.77–2.52). The probability of having health literacy also increased with the increase in the promotion level (*P* < 0.001; Table 2).

**Table 2:** Correlation between the TCM cultural ambiance and TCM health literacy.

**Cultural ambiance**	**Model 1**	**Model 2**	***P* for trend**
**Continuous Health Literacy score**, β **(95%CI)**			
Low	Ref	Ref	< 0.001
Medium	7.92 (7.08, 8.76)	5.96 (5.19, 6.73)	
Better	11.09 (10.45, 11.72)	9.75 (9.16, 10.33)	
High	20.09 (19.32, 20.86)	11 (10.24, 11.76)	
**Qualification of Health Literacy, OR (95%CI)**			
Low	Ref	Ref	< 0.001
Medium	2.11 (1.77, 2.52)	1.76 (1.46, 2.12)	
Better	2.50 (2.16, 2.89)	2.38 (2.05, 2.77)	
High	7.28 (6.27, 8.45)	3.27 (2.79, 3.84)	

Model 1 is a crude model, and model 2 is adjusted for age, sex, education level, occupation, and annual income.

### 3.3 Correlation between the TCM cultural ambiance, TCM health literacy, and self-health evaluation

Referring to grade 0 of the promotional ambiance as a baseline, no statistically significant correlation was found between grade 1 and self-health evaluation (β = 0.21, 95% CI: −0.26 to 0.67). However, the average self-health evaluation score increased by 1.47 points at grade 2 (β = 1.47, 95% CI: 1.12–1.83) and by 4.61 points at grade 3 (β = 4.61, 95% CI: 4.19–5.04). These findings were consistent even after adjusting for age, sex, and five other variables. TCM health literacy was associated with self-health evaluation (β = 0.06, 95% CI: 0.05–0.07). Individuals with TCM health literacy had an average increase of 2.1 points in their self-rated health score compared with those without TCM health literacy (β = 2.10, 95% CI: 1.72–2.48). After adjusting for covariates, an average increase of 0.80 points was noted (β = 0.80, 95% CI: 0.40–1.19) (Table 3).

**Table 3:** Correlation between the TCM cultural ambiance, TCM health literacy, and self-assessed health, *β* (95% CI).

	**Model 1**	**Model 2**
**Cultural ambiance**		
Low	Ref	Ref
Medium	0.21 (−0.26, 0.67)	−0.23 (−0.69, 0.23)
Better	1.47 (1.12, 1.83)	1.35 (1.00, 1.70)
High	4.61 (4.19, 5.04)	2.74 (2.28, 3.19)
**Health literacy**		
Continuous	0.06 (0.05, 0.07)	0.02 (0.02, 0.03)
< 70	Ref	Ref
≥70	2.1 (1.72, 2.48)	0.80 (0.40, 1.19)

Model 1 is a crude model, and model 2 is adjusted for age, sex, education level, occupation, and annual income.

### 3.4 Correlation between the TCM cultural ambiance, TCM health literacy, and reporting of chronic diseases

Based on grade 0 of the promotional ambiance, the risk of chronic diseases in grade 1 was 0.88 times that of the reference (OR = 0.88, 95% CI: 0.80–0.98). The reporting rate of chronic diseases decreased with the increase in the level of cultural promotion ambiance. After adjusting for covariates, the risk of chronic diseases reported by grade 3 was 0.86 times that of grade 0 (OR = 0.86, 95% CI: 0.76–0.97). Individuals with health literacy were 47% less likely to report chronic diseases compared with those without health literacy (OR = 0.53, 95% CI: 0.48–0.58). After adjusting for age, education level, occupation, and other factors, individuals with health literacy were 10% less likely to report chronic diseases (OR = 0.90, 95% CI: 0.80–1.00) (Table 4).

**Table 4:** Correlation between the TCM cultural ambiance, TCM health literacy, and chronic diseases, OR (95% CI).

	**Model 1**	**Model 2**
**Cultural ambiance**		
Low	Ref	Ref
Medium	0.88 (0.8, 0.98)	1.04 (0.92, 1.17)
Better	1.02 (0.95, 1.1)	1.03 (0.95, 1.13)
High	0.38 (0.34, 0.42)	0.86 (0.76, 0.97)
**Health literacy**		
Continuous	0.98 (0.98, 0.99)	0.99 (0.99, 0.99)
< 70	Ref	Ref
≥70	0.53 (0.48, 0.58)	0.90 (0.80, 1.00)

Model 1 is a crude model, and model 2 is adjusted for age, sex, education level, occupation, and annual income.

### 3.5 Mediating effect of TCM health literacy

**Figure 1:**
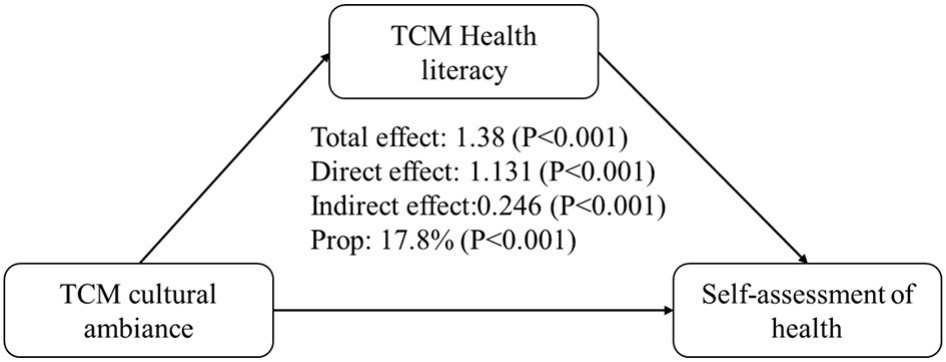
Mediating effect of TCM health literacy on the relationship between the TCM cultural ambiance and self-rated health.

**Figure 2:**
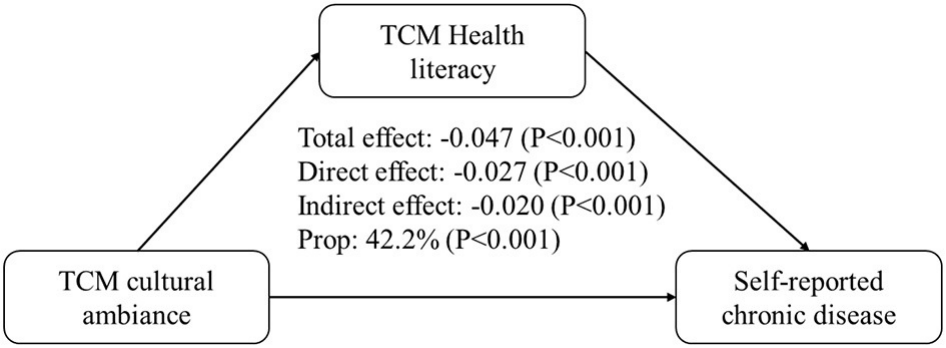
Mediating effect of TCM health literacy on the relationship between the TCM cultural ambiance and the reporting of chronic diseases.

The overall impact of the TCM cultural ambiance on self-health evaluation was 1.38. When TCM health literacy was considered, the direct impact was 1.131 and the indirect impact was 0.246. All pathways were statistically significant (*P* < 0.001), and the indirect impact represented 17.8% of the total impact. The total impact of the promotional ambiance on the reporting of chronic diseases was −0.047, with a direct impact of −0.027 and an indirect impact of −0.020. All pathways were statistically significant (*P* < 0.001), and the indirect impact accounted for 42.2% of the total impact (Table 5, Figures 1, 2).

**Table 5:** Mediator model.

	**X → Y**	**X + M → Y**	**Proportion of mediation (%)**	**R^2^**
**Self-reported health status scores**, β **(95%CI)**				**0.36**
Cultural ambiance			17.8 (14.0, 0.22)	
Low	Ref	Ref		
Medium	−0.23 (−0.69, 0.23)	−0.31 (−0.77, 0.15)		
Better	1.35 (1.00, 1.70)	1.21 (0.85, 1.57)		
High	2.74 (2.28, 3.19)	2.59 (2.12, 3.05)		
Health literacy		0.01 (0.01, 0.02)		
**Self-reported chronic diseases, OR (95%CI)**				**0.31**
Cultural ambiance			42.2 (34.6, 52.0)	
Low	Ref	Ref		
Medium	1.04 (0.92, 1.17)	1.05 (0.94, 1.18)		
Better	1.03 (0.95, 1.13)	1.06 (0.97, 1.15)		
High	0.86 (0.76, 0.97)	0.88 (0.77, 0.99)		
Health literacy		0.9978 (0.9957, 0.9999)		

## 4 Discussion

This study revealed that the TCM health literacy among residents in Jiangsu Province, China, was 14.76%, which was lower than the national average level (22.56%). The prevalence rate of chronic diseases was 32.66%, which was consistent with the prevalence rate of chronic diseases in China in 2018 (34.29%) (21, 22).

In this study, the results revealed a positive correlation between the cultural promotion ambiance and TCM health literacy, as well as direct impacts on self-health assessment and prevalence of chronic diseases. This pertained to a cultural milieu that encompasses the dissemination of cultural knowledge (popularization, comprehension, acceptance, and practice), while also echoed the concept of traditional Chinese medicine health literacy as advanced by scholars like Wang Suzhen and Zhang Xueyan, drawing from the theory of knowledge-belief-action (12). Zhu Bing demonstrated that promotion intervention activities could improve TCM health literacy (16). However, the findings of this study presented a more comprehensive illustration of this conclusion. From the perspective of the promotion level, the higher the degree of promotion, the more pronounced the impact on health literacy.

The principle of this association may be inseparable from the theory of information dissemination. Ge et al. (23) confirmed that exposure to TCM information positively affected the adoption of TCM practices. Moreover, the level of TCM health knowledge, attitude, subjective norms, and perceived behavior control all played a positive mediating role. Huang et al. (24) confirmed the establishment of the publicity–knowledge–attitude–practice pathway through the mediating effect model. Social support could contribute to strengthening the public's cultural identity, fostering belief, enabling conscious and independent decision-making in behavioral patterns, and facilitating the transition from knowledge to belief to action. Li et al. (25) demonstrated that informational support could enhance individuals' belief and promote information-seeking behavior. The promotion of TCM culture served as a means of information support, facilitating the dissemination of knowledge, skills, and health concepts to enable the public to appreciate the cultural legacy and medical significance of TCM. This was conducive to the improvement in public health level.

Additionally, this study indicated that health literacy significantly mediated the relationship between cultural promotion ambiance and self-health evaluation. The findings demonstrated a positive correlation between TCM health literacy and self-rated health, as well as a negative impact on reporting chronic diseases, consistent with prior findings (26–28). Adherence to TCM concepts and healthy behaviors is essential for preserving and enhancing overall wellbeing (29). Throughout the fight against the coronavirus disease 2019 pandemic, TCM has exhibited evident efficacy in managing mild cases and improving cardiopulmonary function, thereby substantially lowering the mortality rate among severe patients (30, 31). Furthermore, TCM s potential enables early intervention and effective treatment for various chronic, severe, and intricate conditions, thereby reducing patient distress and improving their overall wellbeing (32). It is hypothesized that TCM health literacy might impact health outcomes by influencing the adoption of health-related behaviors, serving as a mediating variable. Research has demonstrated a positive correlation between health literacy and health behaviors (33, 34), and health literacy indirectly impacts health status by influencing health behaviors and self-efficacy (34). These findings provided empirical support for the aforementioned hypothesis.

Since the transition from “public Chinese medicine literacy” to “Chinese medicine health literacy,” increasing emphasis has been laid on Chinese medicine health literacy by the Chinese government, academia, and the general population. This shift has resulted in heightened theoretical and social support for the assessment and appraisal of Chinese medicine health literacy, as well as for more extensive and thorough research. Improving TCM health literacy is essential for scientific and technological advancement, economic growth, and preservation and propagation of traditional Chinese culture (35). Research has indicated that access to information, ability to effectively utilize information, and social support are critical determinants influencing the enhancement of health literacy (36, 37). Health education functions as a method for obtaining knowledge, disseminated by the community, medical service institutions, and schools. It serves as a conduit for information dissemination and promotes the recognition and application of information through cooperative learning. As such, it constitutes a promising approach to enhancing health literacy (36, 38). Furthermore, it is essential to integrate traditional media and new media when disseminating information to meet the public's demand for novelty, diversify promotional channels, and expand communication coverage. This approach can facilitate public access to health information, promoting better understanding and engagement with health knowledge (39–41). Besides, it is imperative to promote cooperation among various entities, policies, and social networks to facilitate the integration of TCM into all segments of society. This approach can contribute to enhancing public health knowledge, advocating for health promotion and maintenance, and enhancing overall wellbeing.

### 4.1 Limitations

First, it was a cross-sectional study, which could not confirm the causal correlation among the three factors. The participants might have paid more attention to health knowledge, leading to the Neyman bias. Second, the health status of the population was assessed through a questionnaire survey, introducing information bias. Thirdly, the questionnaire lacks detailed content. For example, in the investigation of occupational information, nearly 35% of individuals reported their occupation as “other.” This to some extent limits further analysis of occupational factors. Fourth, this study only focused on the association between TCM literacy and health, but this may be influenced by various aspects, such as the participants' knowledge of Western medicine, medication literacy, and health literacy. These factors may have complex interactions with TCM literacy, potentially collectively promoting human health. However, this study lacks relevant data to explore these influences. Finally, the definition of cultural promotion ambiance was ambiguous, and the effectiveness of various publicity measures and different audiences might have impacted the outcomes. Hence, future endeavors should include community intervention trials or long-term cohort follow-up studies to assess health status using rigorous scientific measurement tools and indicators. Simultaneously, refining specific health promotion measures to offer more compelling causal evidence is essential.

## 5 Conclusions

This study unveiled the correlation between the TCM cultural ambiance, TCM health literacy, and overall health status. It also illustrated the mediating effect of TCM health literacy on the relationship between the ambiance prmoting TCM culture and individual health levels, introducing a novel concept for devising strategies aimed at further enhancing the wellbeing of the populace. The findings suggested that it was theoretically viable to bolster the health of residents by fostering the TCM culture propaganda, facilitating access to information about TCM culture, broadening the dissemination of promotional efforts, and elevating the level of health literacy pertaining to TCM.

## Data availability statement

The original contributions presented in the study are included in the article/supplementary material, further inquiries can be directed to the corresponding author.

## Ethics statement

The studies involving humans were approved by Ethics Review Committee of the National Administration of Traditional Chinese Medicine. The studies were conducted in accordance with the local legislation and institutional requirements. The participants provided their written informed consent to participate in this study.

## Author contributions

XZ: Conceptualization, Investigation, Resources, Supervision, Writing – original draft. XH: Formal analysis, Methodology, Writing – original draft. ZZ: Conceptualization, Investigation, Project administration, Writing – review & editing.
